# Regulation of cell cycle by the anaphase spindle midzone

**DOI:** 10.1186/1471-2121-5-49

**Published:** 2004-12-23

**Authors:** Maki Murata-Hori, Greenfield Sluder, Yu-li Wang

**Affiliations:** 1 Department of Physiology, University of Massachusetts Medical School, 377 Plantation St., Worcester, Massachusetts, 01605, USA; 2 Mammalian Cell Biology Group, Temasek Life Sciences Laboratory, 1 Research Link, National University of Singapore, 117604, Singapore; 3 Department of Cell Biology, University of Massachusetts Medical School, 377 Plantation St., Worcester, Massachusetts, 01605, USA

## Abstract

**Background:**

A number of proteins accumulate in the spindle midzone and midbody of dividing animal cells. Besides proteins essential for cytokinesis, there are also components essential for interphase functions, suggesting that the spindle midzone and/or midbody may play a role in regulating the following cell cycle.

**Results:**

We microsurgically severed NRK epithelial cells during anaphase or telophase, such that the spindle midzone/midbody was associated with only one of the daughter cells. Time-lapse recording of cells severed during early anaphase indicated that the cell with midzone underwent cytokinesis-like cortical contractions and progressed normally through the interphase, whereas the cell without midzone showed no cortical contraction and an arrest or substantial delay in the progression of interphase. Similar microsurgery during telophase showed a normal progression of interphase for both daughter cells with or without the midbody. Microsurgery of anaphase cells treated with cytochalasin D or nocodazole indicated that interphase progression was independent of cortical ingression but dependent on microtubules.

**Conclusions:**

We conclude that the mitotic spindle is involved in not only the separation of chromosomes but also the regulation of cell cycle. The process may involve activation of components in the spindle midzone that are required for the cell cycle, and/or degradation of components that are required for cytokinesis but may interfere with the cell cycle.

## Background

Microtubules undergo striking reorganization during anaphase and telophase. During anaphase, antiparallel, interdigitating microtubules and many associated proteins become organized into discrete bundles in the spindle midzone [1], the region between separated chromosomes. As the cell enters cytokinesis, these midzone microtubule bundles merge into a single compact, electron-dense structure called midbody. It is generally recognized that, at least for cultured animal cells, midzone microtubules play a major role in cytokinesis. For example, cleavage furrows, both normal and ectopic, are associated with similar microtubule bundles [2-4], while regions physically blocked from midzone microtubules by micromanipulation are unable to undergo cytokinesis [5]. Moreover, continuous interactions of midzone microtubules with the cell cortex are required for sustaining the cytokinesis of cultured animal cells [6].

Recent progress suggested that, in addition to cytokinesis, the spindle midzone might be involved in additional functions. For example, midzone and midbody microtubules are associated with many regulatory proteins apparently unrelated to cortical contraction, such as the DNA replication initiator Orc6 [7], the inhibitor of apoptosis survivin [8], and the tyrosine kinase binder Nir2 [9]. In addition, mother centrioles were found to migrate to the midbody during telophase before returning to their interphase position, possibly activating some centrosomal components for cell cycle progression [10]. Furthermore, treatment of dividing cells with dihydrocytochalasin B, an inhibitor of cytokinesis, caused not only the inhibition of cytokinesis but also G1 arrest after mitosis [11], raising the possibility that ploidy, cortical contraction, and/or activation/deactivation of proteins during cytokinesis, may play a role in the regulation of the following cell cycle.

To address this possibility, we severed cells at anaphase or telophase by microsurgery, to bypass the normal mechanism of cytokinesis. The functional role of the spindle midzone or midbody in the following cell cycle was then tested by manipulating the position of the microsurgery or by applying pharmacological agents. Using extended time-lapse microscopy, we found that anaphase midzone microtubules play an important role in the progression of the subsequent interphase. However, there was no evidence of the involvement of telophase midbody in cell cycle progression.

## Results and discussion

**Figure 1:**
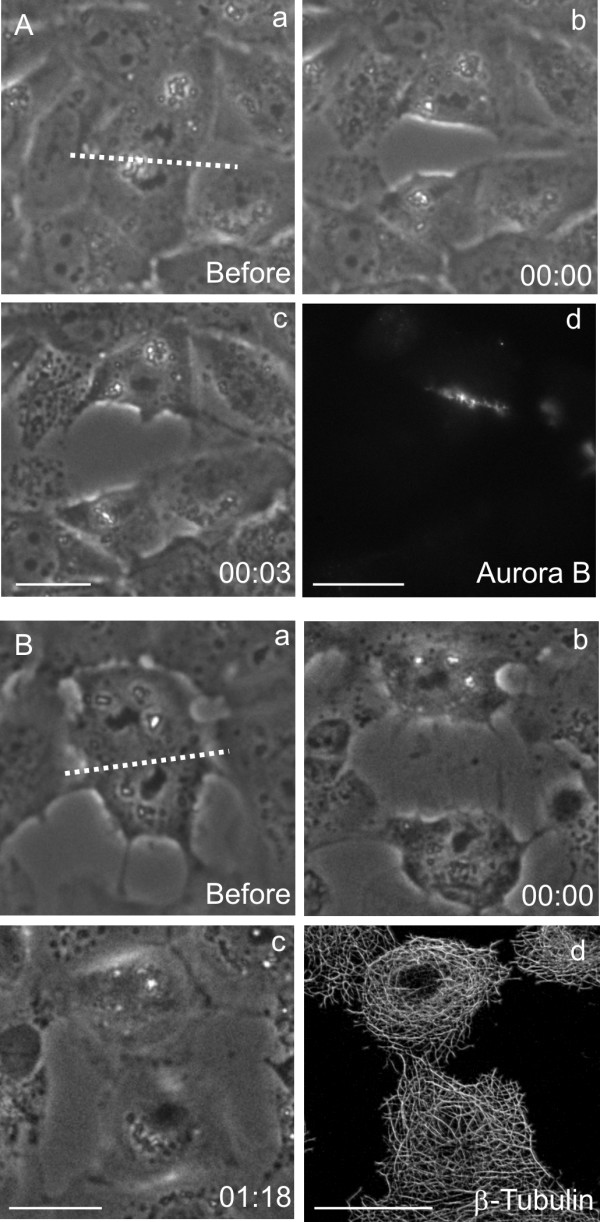
**Microsurgery of dividing NRK cells. **The site of severing is indicated by dotted lines (A, a; B, a). Immunofluorescence indicates that aurora B, a protein associated with midzone microtubules, is partitioned predominantly to the cell with midzone microtubules (A, d). Microtubules, staining of another cells cut in a similar way, showed an indistinguishable network in both daughter cells after recovering for one hour (B, d). Bar, 20 μm.

**Figure 2:**
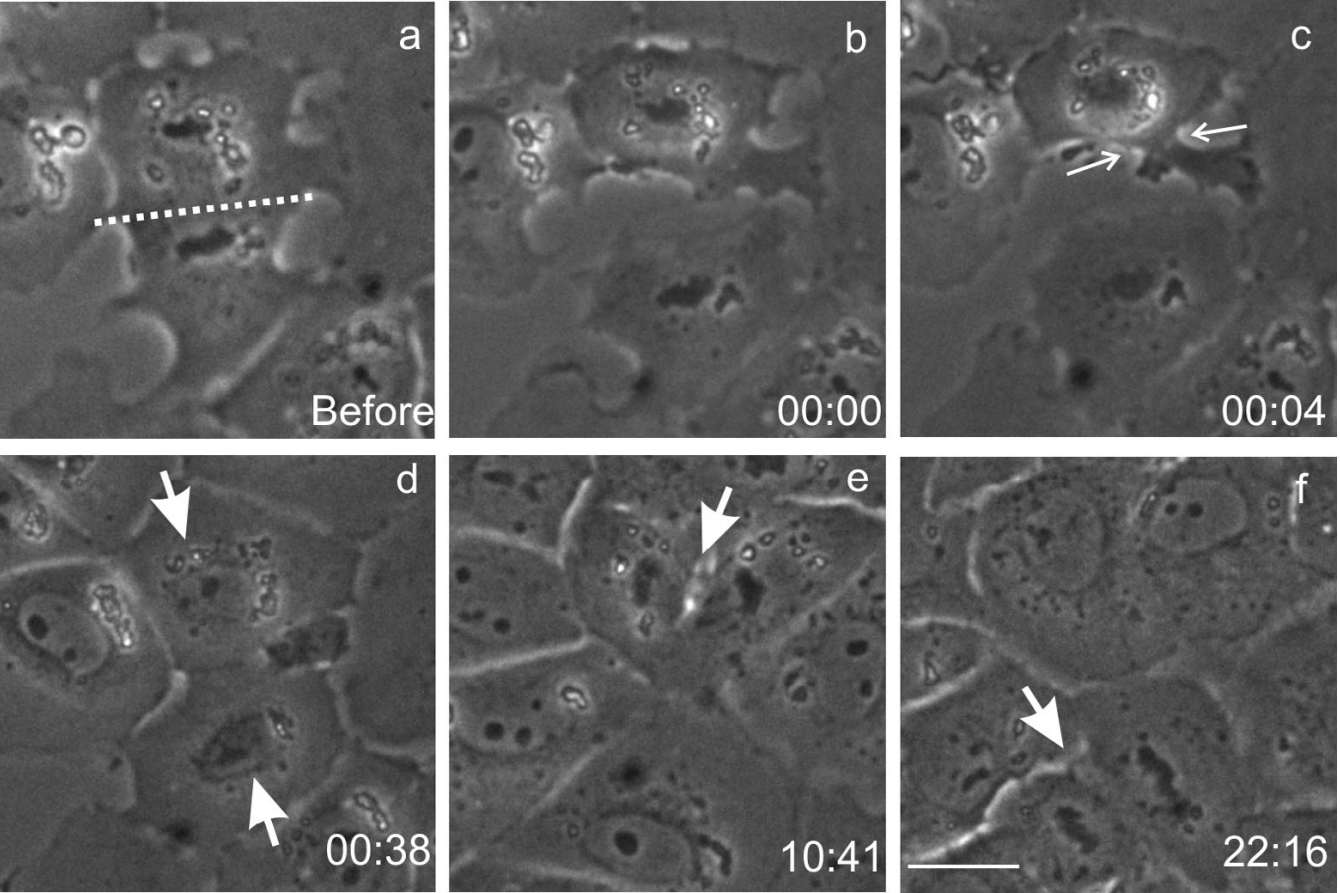
**Inhibition of the interphase progression following surgical removal of the spindle midzone structures. **An NRK cell was cut at anaphase to form daughter cells with (top) and without (bottom) the spindle midzone structures (cutting site indicated by the dotted line, a). Subsequent time-lapse recording indicates that both daughter cells formed nuclear envelop (arrows, d). However, only the daughter cell with spindle midzone (top) showed cytokinesis-like contractions (c, arrows). The daughter cell with spindle midzone entered the subsequent mitosis 11 hours after microsurgery (e, arrow), whereas the daughter cell without spindle midzone entered mitosis 22 h after microsurgery (f, arrow). Time elapsed since cutting is shown in hours:minutes. Bar, 20 μm.

To investigate if anaphase spindle midzone affects interphase progression in daughter cells, we cut NRK52E cells at early anaphase, as soon as sister chromosomes have separated completely, with a fine glass fiber to form daughter cells with or without spindle midzone structures. Staining of severed daughter cells with antibodies against aurora B (Figure 1A), which is known to relocate from centromeres to the spindle midzone during early anaphase [12,13], confirmed that the spindle midzone was removed from one of the daughter cells. However, both daughter cells showed typical interphase microtubules organization (Figure 1B), and normal cellular and nuclear morphology (Figure 2, d), indicating that cells recovered fully from the microsurgery and exited mitosis successfully.

A total of eight manipulated cells were followed by extended time-lapse imaging through the following interphase. All the daughter cell with spindle midzone showed cytokinesis-like cortical contraction activities, while the daughter cell without spindle midzone showed no cortical contraction (Figure 2, see Additional file 1). The daughter cell with spindle midzone subsequently progressed with normal timing through the following interphase, entering mitosis at a time similar to that of adjacent control cells (Figure 2, Table 1). However, in 5 out of 8 cases, the cell without midzone showed a duration of interphase more than twice that of the sister cell with spindle midzone (Table 1). In the example shown in Figure 2, interphase lasted for 22 h, as compared to the normal 11 h for the sister cell with the spindle midzone. In two cases, cell cycle appeared to be arrested in interphase since no mitosis was observed over a period of 34 – 46 h.

**Table 1:** Duration of interphase for daughter cells with and without the spindle midzone or midbody^#^

	midbody/spindle midzone	
	+	-
spindle midzone (8)	721 ± 66	1444 ± 236**
spindle midzone (5)*	671 ± 28	1724 ± 301**
midbody (9)	610 ± 92	678± 113
control*** (66)	611 ± 16	

^#^Average time in minutes ± SEM

*Average of 5 experiments that showed at least a doubling in the length of interphase for the daughter without the midzone.

**In 3 cases, the daughter cell without spindle midzone failed to enter mitosis during the period of time-lapse recording. For these cells, the length of interphase was assumed to equal the length of observation. Therefore, this average value represents an under-estimate.

***Two unmanipulated cells near the manipulated cell were measured in each experiment as controls.

**Figure 3:**
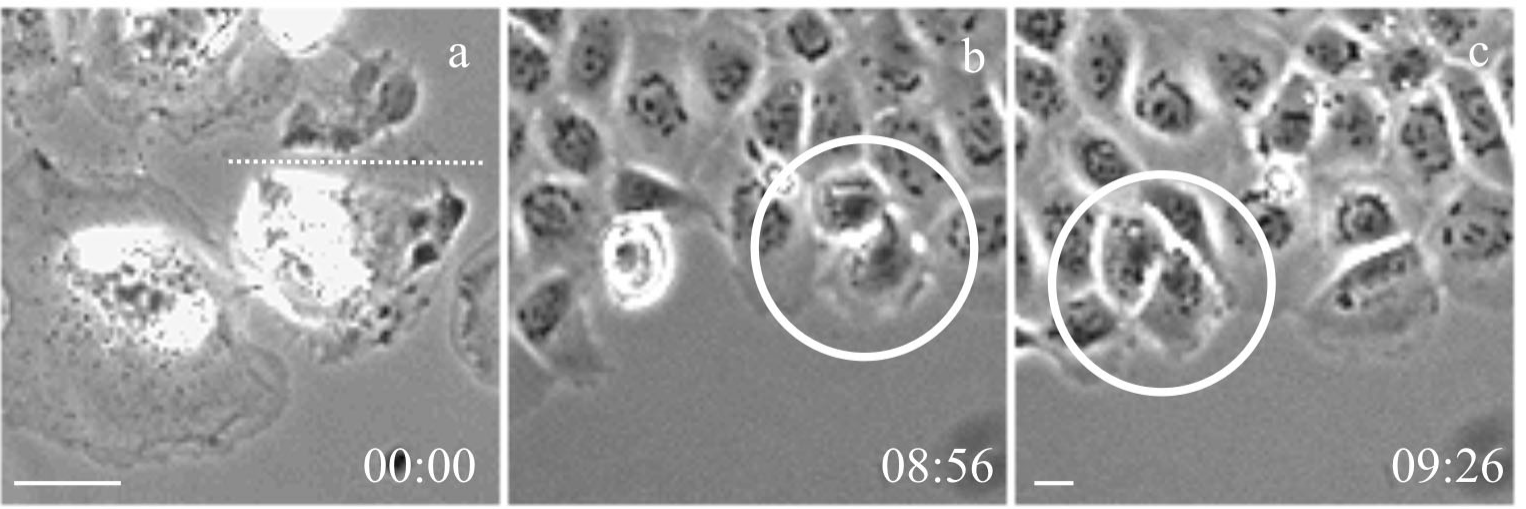
**Normal interphase progression following manipulation of the size of a daughter cell. **A lateral region near the pole was cut away from one of the daughter cells at the end of cytokinesis (cutting site indicated by the dotted line, a). Subsequent time lapse imaging revealed a normal interphase duration in both daughter cells (b, c). Time elapsed since cutting is shown in hours:minutes. Bar, 20 μm.

The inhibition of interphase progression was affected by the position of severing. When cells were severed near the equator to divide midzone structures between the two daughter cells, both cells showed cytokinesis-like cortical contractions and no effect on the progression of interphase. This observation was repeated with 4 cells. To test the possibility that the duration of interphase was affected by the size of the daughter cell, we took pairs of normally divided daughter cells and cut away a lateral region near the pole from one of the daughter cells. In all 5 cases, both daughter cells entered the subsequent mitosis with a timing similar to that of neighboring unmanipulated cells (Figure 3). Together, these results indicate that the progression of interphase was affected directly or indirectly by the presence of the anaphase spindle midzone.

To test if cortical contraction is the primary determining factor of the rate of interphase progression, we treated cells with cytochalasin D at early anaphase for 10 min then severed them at various positions in the spindle midzone, at a time when midbody started to form and ingression started to appear in control cells. Cytochalasin D was removed 20 min later to ensure that there was no lingering cytokinesis activity upon removal of the drug [[14] see Methods]. In all 5 cases, both daughter cells entered the subsequent mitosis with a timing similar to that of neighboring cells, despite the complete inhibition of cortical contraction (Table 2). In addition, unmanipulated, binucleated cells, which failed cytokinesis spontaneously, also entered subsequent mitosis with a similar timing. Thus, the present observation is distinct from the "tetraploidy checkpoint", which was identified with dihydrocytochalasin B-treated REF52 cells [11], but remained controversial with regard to its universal existence [15].

**Table 2:** Duration of interpahse for cells severed in the presence of cytoskeletal inhibitors^#^

	Severed cells^##^	control^###, ##^
cytochalasin D	599 ± 49 (5)	587 ± 24 (10)
nocodazole	892 ± 71 (7)	621 ± 30 (14)

^#^Average time in minutes ± SEM

^##^ Statistics was calculated by grouping the daughter cells. Therefore, the number of daughter cells analyzed was twice the number of experiments as indicated in the parentheses.

^###^Two unmanipulated, dividing cells near the manipulated cell were followed in each experiment as controls.

**Figure 4:**
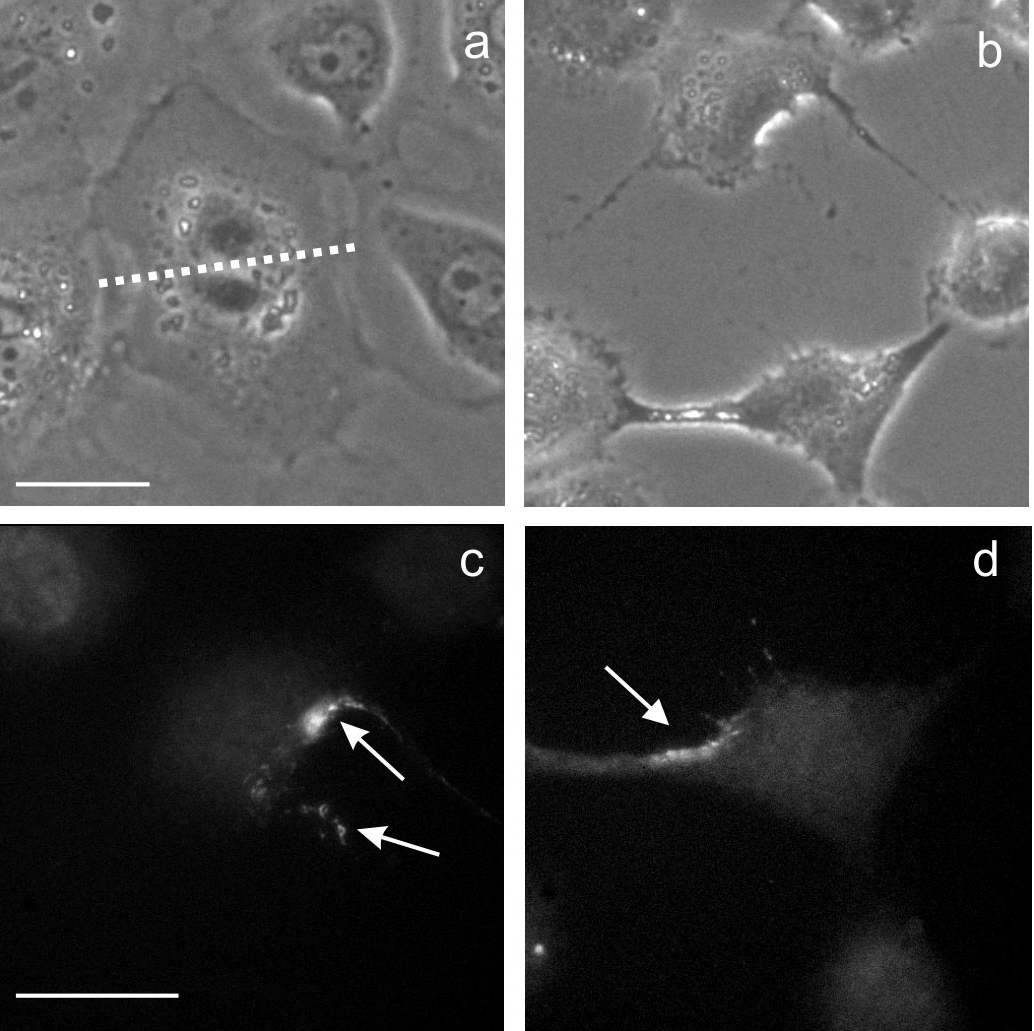
**Presence of aurora B in both daughter cells following microsurgery of nocodazole-treated cells. **An NRK cell at early anaphase was treated with 10 μM nocodazole for ~10 min before microsurgical cut between segregated chromosomes (cutting site indicated by the dotted line, a). Then the daughter cells were released from nocodazole by washing twice with fresh medium. Immunofluorescence of aurora B showed that both daughter cells contained dot-like structures of aurora B along the cell cortex (c, d, arrows). Bar, 20 μm.

We then asked if midzone microtubules, or proteins associated with the anaphase spindle midzone, are involved in interphase progression. Treatment of cells with nocodazole for 6–13 min at early anaphase before severing, with or without additional incubation with the drug, caused a significant delay (~4.5 h; p = 0.002) in the progression of interphase for both daughter cells, as compared to neighboring cells (Table 2). Aurora B in these cells showed a complete dispersal from the midzone (Figure 4) [12], with a similar amount distributed in the two severed cells. These observations suggest the midzone microtubules may provide a scaffold for the activation/deactivation of some components to allow normal progression of the cell cycle. We suspect that the weaker effect compared to that caused by severing may be due to the known resistance of some midzone microtubules to nocodazole [6]. Alternatively, a brief association of the components with the midzone microtubules before and during nocodazole treatment may be sufficient for partial activation/deactivation.

**Figure 5:**
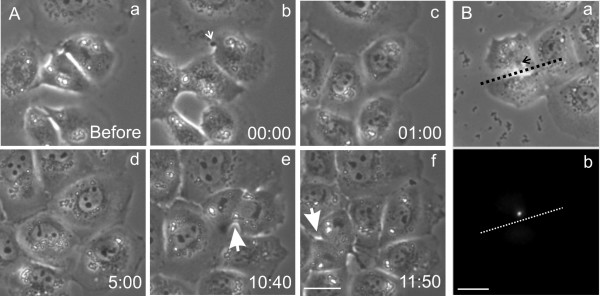
**Normal interphase progression following removal of the midbody. **A cell was cut at the end of cytokinesis to form daughter cells with or without the midbody (A, b, arrow). Subsequent long-term time-lapse imaging indicated a normal phase morphology for both cells (A). The cell with midbody entered mitosis 10 h 40 min after cutting, whereas the cell without midbody entered mitosis ~1 h afterwards (e, f, large arrows). Fluorescence imaging of aurora-B-GFP, a midbody component, confirmed that the midbody is completely segregated into one of the daughter cells (B; cutting indicated by dotted lines) Bar, 20 μm.

We also tested if telophase midbody is required for interphase progression. Cells were severed at mid- or late cytokinesis to form daughter cells with and without the midbody (Figure 5, see Additional file 2). Using cells expressing aurora B-GFP, which is known to associate with the midbody [12,13,16], we confirmed that the midbody was completely segregated from one of the daughter cells (Figure 5B). All nine manipulated pairs showed a normal progression of interphase indistinguishable from that of unmanipulated control cells, irrespective of the presence of the midbody (Table 1). We conclude that the presence or absence of telophase midbody no longer affects the progression of subsequent interphase, despite the similarity of molecular components to those in the earlier spindle midzone. Thus, most likely it is transient catalytic reactions in the anaphase midzone that are crucial for the progression of cell cycle.

Some of major mitotic regulators are degraded by anaphase promoting complex/cyclosome (APC) during anaphase [17]. Degradation of the polo-like kinase (Plk1) and aurora A by APC occurred while they were localized along midzone microtubules [17], raising the possibility that interphase progression may require the degradation of some mitotic/cytokinetic proteins, which may then cause activation of downstream components crucial for cell cycle. In addition, some molecules associated with midzone microtubules may be directly involved in cell cycle events such as DNA synthesis, as suggested by the chromosomal passenger protein-like dynamics of a DNA replication initiating factor, Orc6 during cell division [7].

## Conclusions

Our results suggest that anaphase midzone not only play a role in the stimulation of cytokinesis in cultured cells, but also provide a scaffold for the activation/deactivation of factors essential for the progression of subsequent cell cycle.

## Methods

### Cell culture, microscopy, and image processing

Normal Rat Kidney epithelial cells (NRK-52E; American Type Culture Collection, Rockville, MD) were cultured in Kaighn's modified F12 (F12K) medium supplemented with 10% FBS (JRH Bioscience, Lenexa, KS), 50 U/ml penicillin, and 50 μg/ml streptomycin, on glass chamber dishes as previously described [18]. The cells were maintained at 37°C in a stage incubator built on top of a Zeiss Axiovert S100TV or an Axiovert 35 inverted microscope (Carl Zeiss, Thornwood, NY), and viewed with 10X, NA 0.25 Achrostigmat, 40X, NA 0.75 Plan-Neofluor or 100X, NA 1.30 Plan-Neofluor lens. All images were acquired with a cooled charge-coupled device camera (ST133 controller and CCD57 chip; Roper Scientific, Trenton, NJ) and processed with custom software for background subtraction.

### Microsurgery and drug treatment

Glass needles for microsurgery were prepared with a David-Kopf Model 700 vertical puller. The tip of the needle was melted and elongated into a fine fiber with a Narishige microforge (Model MF900). Microsurgery of the cells was achieved by carefully lowering a fiber onto the target cell followed by slow dragging with a micromanipulator (Leica, Deerfield, IL).

Cytochalasin D (Sigma, St. Louis, MO) were stored at -20°C as 2.5 mM stock in DMSO, and diluted into warm medium before application to cells. We found that treatment of early anaphase cells with cytochalasin D for 30 min completely inhibited cytokinesis even upon removal of the drug, similar to what was reported with dihydrocytochalasin B [14]. Thus, early anaphase cells were treated with cytochalasin D at a final concentration of 2 μM for 10 min before microsurgery. The cells were then incubated for additional 20 min and washed at least twice with fresh medium. Nocodazole (Sigma, St. Louis, MO) was stored at -20°C as 10 mM stocks in DMSO and was diluted with warmed medium before use. Early anaphase cells were incubated with nocodazole for 6–13 min and then cut into two daughter cells with microsurgery. Immediately after microsurgery or following 50 min incubation, the daughter cells were released from nocodazole by incubating with two changes of fresh medium for at least 5 min each. The significance of these results was assessed using analysis of variance (ANOVA) and t-test in Microsoft Excel.

### Transfection and immunofluorescence

Aurora B-GFP was constructed and transfected into NRK cells as described previously [12]. Immunofluorescence of tubulin and reconstruction of microtubules images were carried out as described previously [19]. For immunofluorescence, cells were rinsed with warm cytoskeleton buffer and fixed with 4% paraformaldehyde (EM Science, Gibbstown, NJ) in warm cytoskeleton buffer for 10 min [6]. They were then rinsed thoroughly in the cytoskeleton buffer and permeablized by incubation with 0.5% Triton X-100 in cytoskeleton buffer for 5 min. Fixed cells were rinsed with the cytoskeleton buffer, blocked for 10 min with 1% BSA (Boehringer Mannheim, Indianapolis, IN) in PBS, then incubated with anti-AIM-1 monoclonal antibodies (BD Biosciences, San Jose, CA) at a dilution of 1:200 in PBS with 1% BSA for 45 min at 37°C. After washing with PBS/BSA thoroughly, cells were incubated with Alexa 546-conjugated goat anti-mouse antibodies (Molecular Probes, Eugene, OR) at a dilution of 1:100 for 30 min at 37°C.

## Authors' contributions

MMH carried out all the experimental work and drafted the manuscript. GS and YLW participated in its design and coordination of the research and edited the manuscript. All authors read and approved the final manuscript.

## Supplementary Material

Additional File 1 Interphase progression of the daughter cells with and without spindle midzone. An NRK cell was severed at early anaphase to form daughter cells with and without spindle midzone. Subsequently, time-lapse imaging was performed to investigate if anaphase spindle midzone was involved in interphase progression. The daughter cell with spindle midzone showed a cytokinsis-like cortical contraction and entered into the subsequent mitosis 11 h 23 min after microsurgery, while the daughter cell without spindle midzone showed no cortical contraction and entered into mitosis 23 h 8 min after microsurgery. Click here for file

Additional File 2 Interphase progression of the daughter cells with and without midbody. An NRK cell was severed at telophase to form daughter cells with and without midbody. Subsequently, time-lapse imaging was performed to investigate if midbody was involved in interphase progression. The daughter cell with midbody entered into the subsequent mitosis 7 h 35 min after microsurgery, while the daughter cell without midbody entered into mitosis 9 h 25 min after microsurgery. Click here for file
